# Mechanically Robust and Flame-Retardant Superhydrophobic Textiles with Anti-Biofouling Performance

**DOI:** 10.1021/acs.langmuir.2c02248

**Published:** 2022-10-14

**Authors:** Jie Liu, Yuling Sun, Rui Ma, Xiaoteng Zhou, Lijun Ye, Volker Mailänder, Werner Steffen, Michael Kappl, Hans-Jürgen Butt

**Affiliations:** † Max Planck Institute for Polymer Research, Ackermannweg 10, D-55128 Mainz, Germany; ‡ The Second Clinical Division of Peking University School and Hospital of Stomatology, Anlilu 66, 100101 Beijing, China; § Department of Dermatology, University Medical Center of the Johannes Gutenberg-University Mainz, Langenbeckstr. 1, 55131 Mainz, Germany

## Abstract

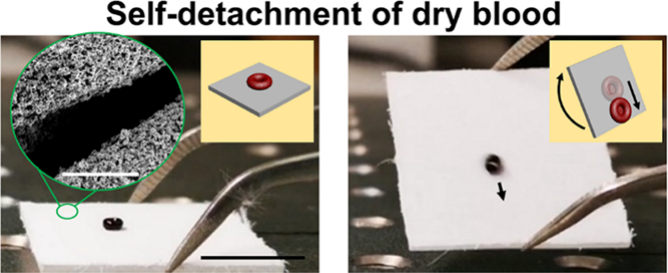

The attachment of bio-fluids to surfaces promotes the transmission of diseases. Superhydrophobic textiles may offer significant advantages for reducing the adhesion of bio-fluids. However, they have not yet found widespread use because dried remnants adhere strongly and have poor mechanical or chemical robustness. In addition, with the massive use of polymer textiles, features such as fire and heat resistance can reduce the injuries and losses suffered by people in a fire accident. We developed a superhydrophobic textile covered with a hybrid coating of titanium dioxide and polydimethylsiloxane (TiO_2_/PDMS). Such a textile exhibits low adhesion to not only bio-fluids but also dry blood. Compared to a hydrophilic textile, the peeling force of the coated textile on dried blood is 20 times lower. The textile’s superhydrophobicity survives severe treatment by sandpaper (400 mesh) at high pressure (8 kPa) even if some of its microstructures break. Furthermore, the textile shows excellent heat resistance (350 °C) and flame-retardant properties as compared to those of the untreated textile. These benefits can greatly inhibit the flame spread and reduce severe burns caused by polymer textiles adhering to the skin when melted at high temperatures.

## Introduction

The adherence of bio-fluids to surfaces is of high concern under medical conditions.^1−3^ Surfaces such as clothes, masks, bandages, and wound dressings contaminated by body-fluids increase the risk of bacterial or viral spread such as COVID-19.^4−11^ Therefore, reducing the adhesion of body fluids to surfaces is one question that should be urgently solved.^12−14^ Superhydrophobic surfaces avoid liquid adhesion by significantly reducing the real contact area.^15,16^ The combination of their intermittent structure and low surface energy supports a stable air cushion between the liquid and surface, which leads to the so-called Cassie state.^15,17^ In view of this state-of-the-art recipe, superhydrophobic surfaces present an effective way for blood repellency by reducing effective contact and interaction area for adsorption of, e.g., proteins and cells.^18−21^ In practice, many surfaces such as wound dressing have contact with blood for a long time, during which the blood becomes dry. Changing wound dressings for injured persons is always an essential procedure to maintain the wound clean and inhibit re-infection. Strongly attached to, e.g., a hydrophilic wound dressing, the formed dry blood could peel off from the wound. Injured people have to suffer secondary pain during the change of wound dressings. Recently, several superhydrophobic textiles were reported to display outstanding blood-repellent performance by reducing blood adhesion and achieving rapid hemostasis.^22,23^ However, research about surfaces with low adherence to dried blood is still scarce.

Another challenge is that textiles used for clothes and soft furnishings are highly combustible. Moreover, textiles composed of polymers melt at a high temperature; this leads the textiles to easily attach to the skin and causes serious burns. For fire safety considerations, people must be rigorous in the selection of fire-retardant furniture, which causes a lot of inconvenience. Inspired by the combustion–inhibition effect of some reported superhydrophobic coatings,^24−26^ textiles with both anti-bio-fluid adhesion and flame-retardant performances will have bright application prospects.

## Results and Discussion

### Micro/Nanostructure, Wettability, and Blood-Repellent Performance of the Textile

**Figure 1:**
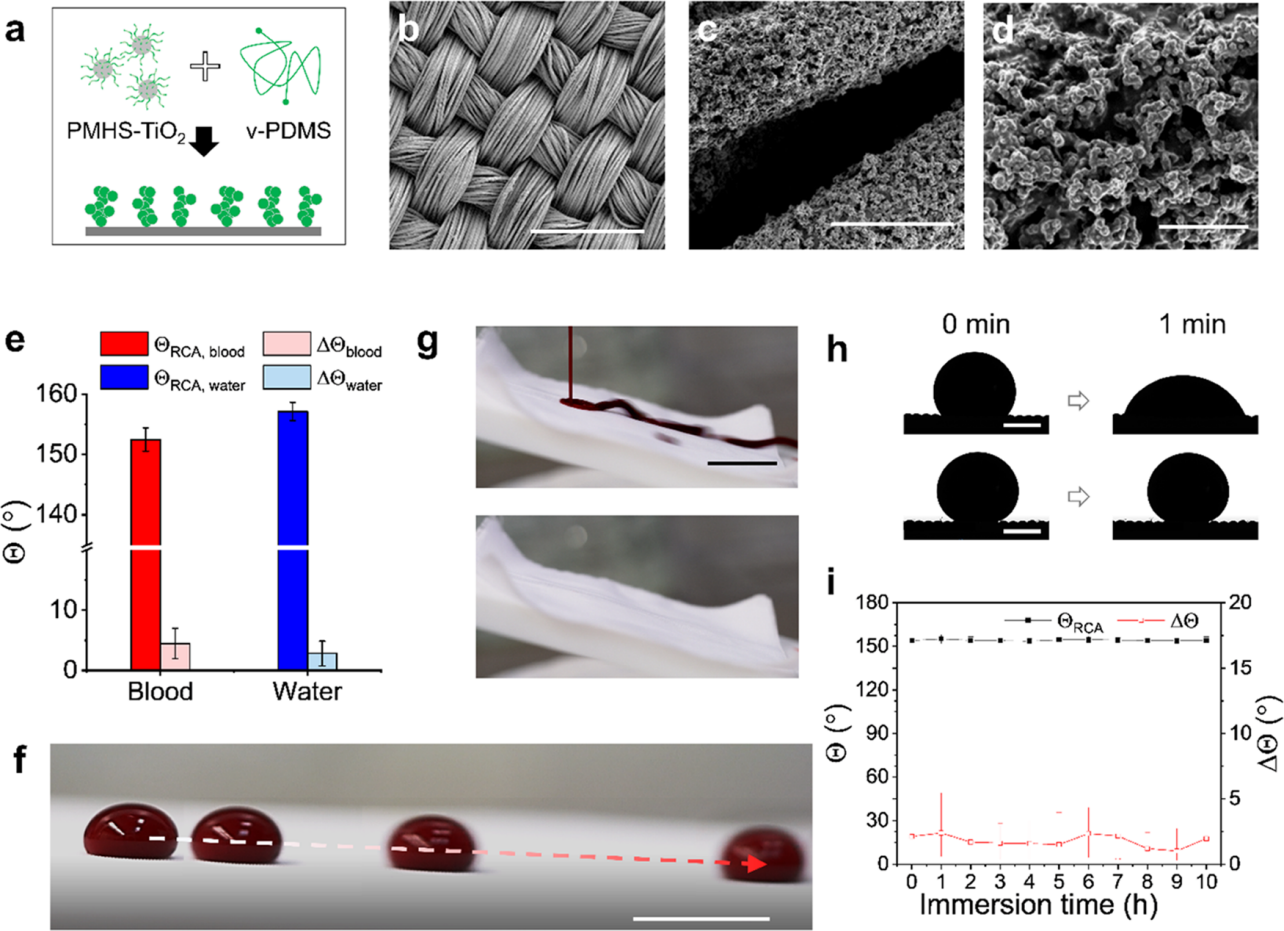
Blood repellency of the TiO_2_/PDMS-coated textile. (a) Schematic illustration of the preparation of the TiO_2_/PDMS nanostructure on the surface of textile fibers. TiO_2_ nanoparticles were coated with PMHS beforehand. (b–d) Scanning electric microscope (SEM) images show the morphology of polyester fabrics coated with the TiO_2_/PDMS nanostructure with increasing magnification from (b to d). Scale bar (from (b to d)): 200, 10, 2 μm. (e) Receding contact angles (Θ_RCA_) and contact angle hysteresis (ΔΘ = Θ_ACA_ – Θ_RCA_, Θ_RCA_: receding contact angle) of blood and water on the superhydrophobic textile. (f) Overlapping digital images show sliding of a blood drop (100 μL) on a tilted surface (α = 2°). Scale bar: 1 cm. (g) Blood flow on the coated textile does not leave stain. Scale bar: 2 cm. (h) Images show the evolution of the shape of a sessile blood droplet on the polyester textile (up) and the TiO_2_/PDMS-coated textile (bottom), respectively. Scale bar: 1 mm. (i) Receding contact angle (red) and contact angle hysteresis (black) of water on the TiO_2_/PDMS-coated textile after being immersed in human blood for different times.

Till now, most superhydrophobic surfaces have low surface energy by surface modification of fluorosilanes. Ongoing regulations and environmental concerns about using highly fluorinated substances have led to continuous research and development of substitutes for use in medical and other life science applications.^27^ Here, we describe a method for fabricating blood-repellent, flame-retardant, and fluorine-free textiles. The method is based on our recently described superhydrophobic coating composed of titanium dioxide (TiO_2_) nanoparticles and polydimethylsiloxane (PDMS).^23^ The coating was found to exhibit superhydrophobic, photocatalytic active, antibacterial, and fast-hemostatic performances. Therefore, a polyester textile was coated with the TiO_2_/PDMS nanostructure by rinsing in a mixture of vinyl-terminated polydimethylsiloxane (V-PDMS), polymethylhydrosiloxane-modified-TiO_2_ (PMHS-TiO_2_), and silicone oil (SO, viscosity: 10 cSt) with *m*_PMHS-TiO_2__/*m*_V-PDMS_ = 0.1*, V*_SO_/*V*_V-PDMS_ = 3 (Figure 1a–d). The PMHS-TiO_2_ was pre-prepared by ultraviolet (UV) illumination of the mixture of TiO_2_ nanoparticles and PMHS. The reaction was allowed to proceed at 60 °C for 6 h. After removing the unreacted PDMS and SO, a superhydrophobic surface with hierarchical structures formed. The hierarchical surface composed of microscale fiber and the TiO_2_/PDMS nanostructure showed high receding contact angles (Θ_blood,RCA_ = 153°, Θ_water,RCA_ = 157°) and low contact angle hysteresis (ΔΘ_blood_ = 4°, ΔΘ_water_ = 3°) (Figure 1e) toward blood and water. Blood drops (100 μL) easily slide on the coated textile at a low tilt angle (α = 2°) (Figure 1f). In addition, blood flowing over the TiO_2_/PDMS-coated textile did not leave any stain on the surface (Figure 1g). This indicates that the textile can efficiently reduce blood contamination by wet adherence.

The penetration and spreading of blood on the textiles will increase the blood adhesion strength and contamination area. The TiO_2_/PDMS-coated textile effectively blocks blood penetration and spread into the textile by its superhydrophobicity. A sessile blood droplet (5 μL) on the TiO_2_/PDMS-coated textile maintains a spherical shape without penetrating into the textile. In contrast, the blood droplet on the uncoated polyester textile surface penetrates easily and rapidly into the pores between the fibers, indicated by the decreasing contact angle with time (Figures 1h and S1). This inhibits the attachment of blood drops on the surface and helps to stop the bleeding, e.g., a pressure bandage. Even after immersing the textile in blood for 10 h (Figure 1i), the textile demonstrates unchanged superhydrophobicity with Θ_water, RCA_ > 150° and ΔΘ_water_ < 5°. Thus, the air cushion between the coated textile and blood is stable enough to ensure the Cassie state of liquid blood on the surface.

### Low Adhesion of Dry Blood on the Textile

**Figure 2:**
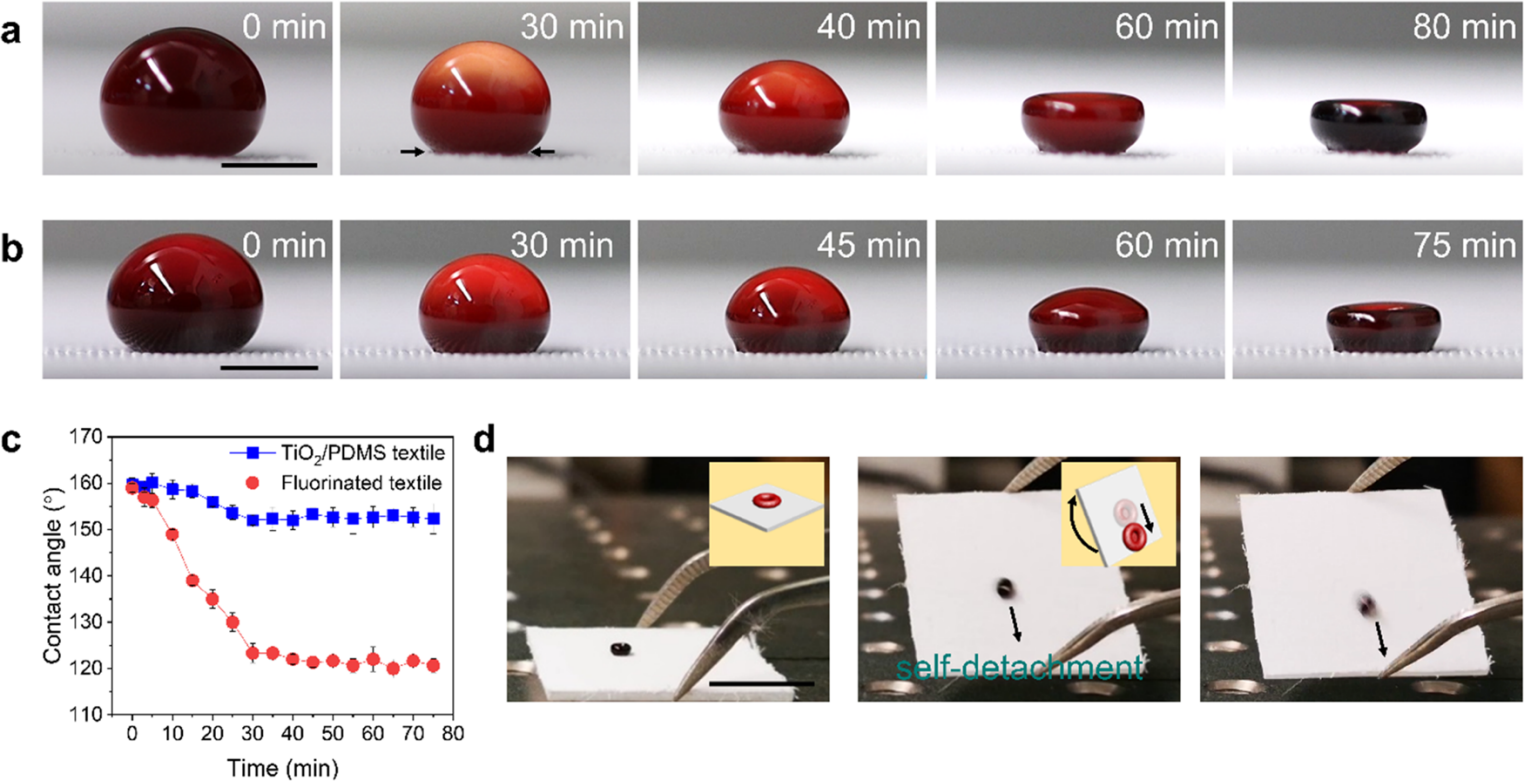
Evaporation of a blood drop on the TiO_2_/PDMS-coated textile. (a) Side-view images show the drying process of a blood drop (20 μL) on the TiO_2_/PDMS-coated textile. Scale bar: 2 mm. (b) Side-view images show the drying process of a blood drop (20 μL) on the fluorinated textile. Scale bar: 2 mm. (c) Contact angles of the blood drops evaporating on the TiO_2_/PDMS-coated textile and the fluorinated textile. (d) Images show self-detachment of dried blood under gravity by tilting the surface. The volume of the original blood was 20 μL. Scale bar: 5 mm.

Due to the stable air layer trapped on the surface and the resulting small real contact area, the contact adhesion of dry blood on the TiO_2_/PDMS coated textile is weak. To further demonstrate the low adhesion, a 20 μL of human blood drop was deposited and dried on the textile (Figure 2a). While drying, the three-phase contact line retracted from 0 to 40 min. With progressing evaporation, the blood cells coagulated at the liquid-vapor interface of the lower part of the blood drop, which caused the interface to gradually harden and become immobile. This results in a stratification; red blood cells sediment from the top to the bottom, indicated by the gradient in color (*t* = 30 and 40 min). As a result, the bottom part of the drop stiffens and no longer follows the shrinking process (40–80 min). The shape of the blood drop changed from an asymmetric ellipse to a bowl-like structure with two high sides and a low center during the whole drying process (Figure S2). A similar evaporation process of the blood drop was observed on the fluorinated textile modified with 1*H*,1*H*,2*H*,2*H*-perfluorooctyltrimethoxysilane (Figure 2b). The blood drop displayed a spherical shape when placed on a fluorinated textile. However, the superhydrophobicity of the surface was not stable without nanostructures. The pinning of the three-phase contact line of the blood drop appeared on the fluorinated textile at the beginning of the evaporation.

The contact angles of blood drops were traced during the evaporation process on textiles (Figure 2c). On the TiO_2_/PDMS-coated textile, the contact angle was larger than 150° during the whole drying process. The contact angle of the dry blood at 75 min was 152° ± 3°. In contrast, on the fluorinated textile, the contact angle of the blood drop decreased from 159° ± 1° at 0 min to 123° ± 2° at 30 min. Thus, the drying blood drop adheres more strongly to the fluorinated textile than that to the TiO_2_/PDMS-coated textile. Adhesion of the dry blood to the TiO_2_/PDMS-coated textile is so low that it detaches spontaneously under gravity when tilting the textile (Figure 2d).

**Figure 3:**
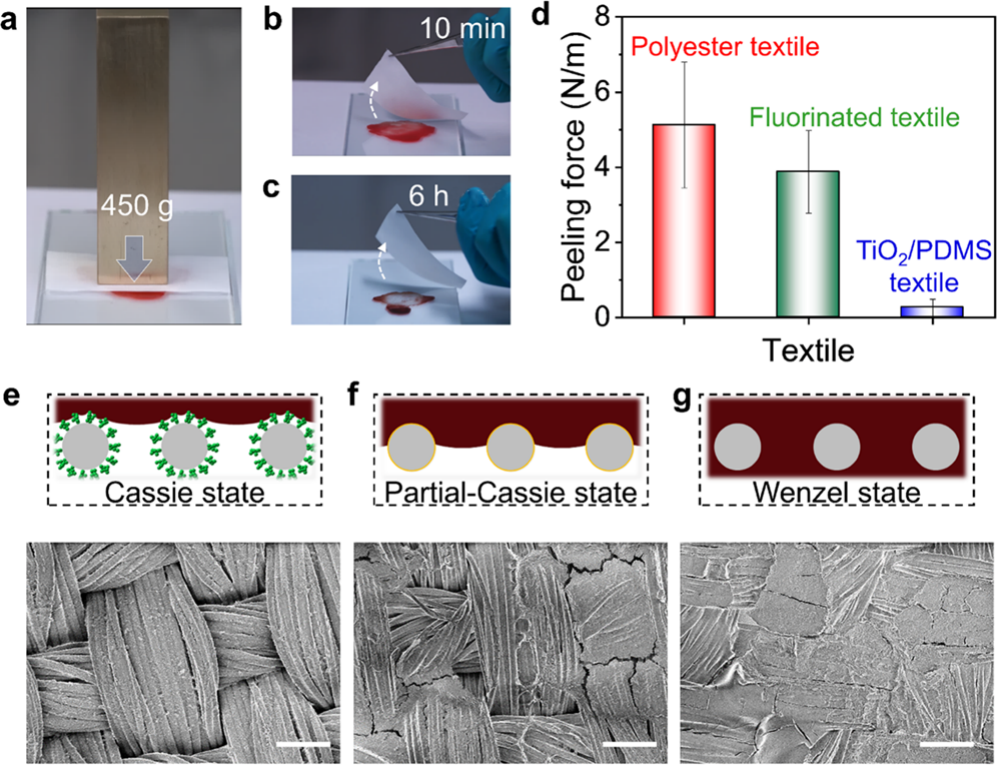
Low adhesion of dry blood on the TiO_2_/PDMS-coated textile. (a) Drying process of blood on textiles under a pressure of a 450 g copper block. (b) Peeling of the TiO_2_/PDMS-coated textile on wet blood. (c) Peeling of the TiO_2_/PDMS-coated textile on dry blood. (d) Peeling forces between the textiles and dry blood. The textiles includes the TiO_2_/PDMS-coated textile, fluorinated textile, and hydrophilic polyester textile. (e–g) SEM images show blood residues attached to the TiO_2_/PDMS-coated textile (e), fluorinated textile (f), and polyester textile (g) after peeling from dry blood. Scale bar: 100 μm. Corresponding schemes illustrate the interfaces between dry blood and textiles. The dark red parts represent the dry blood, the gray circles represent textile fiber, the green structures represent the TiO_2_/PDMS nanostructure, and the orange lines represent the fluorination coating on the textile fiber.

The peeling adhesion of the dry blood on the TiO_2_/PDMS-coated textile was measured. A 7.2 kPa pressure was loaded on top of the textile in the blood for a certain time (Figure 3a). When peeling the textile after 10 min, during which time the blood was in wet state, nothing was left on the superhydrophobic textile (Figure 3b). The blood was completely dry 6 h later, and no stain was observed on the TiO_2_/PDMS-coated textile after peeling (Figure 3c). We further measured the force (*F*_peeling_) needed to peel off the textiles from dry blood (Figure 3d). The peeling force of the TiO_2_/PDMS-coated textile (*F*_peeling_ ≈ 0.26 N/m) on dry blood was reduced to around 20 times lower than the original, uncoated polyester textile (*F*_peeling_ ≈ 5.13 N/m) and 15 times lower than the fluorinated textile (*F*_peeling_ ≈ 3.88 N/m). It can hence be recognized that the coated fabric exhibits simultaneously low adhesion to wet and dry blood with high pressure resistance.

We attribute the low adhesion of blood on the TiO_2_/PDMS-coated textile to the fact that it is in the Cassie state and has a very small contact area with the surface (Figure 3e). The hierarchical micro/nanostructure significantly decreases the contact area between blood and the textile. On the hydrophobic fluorinated textile without nanostructures, the microscale fibers can only partially support the blood with a metastable air layer (Figure 3f). Blood will partially transit to the Wenzel state by penetrating between the polyester fibers under external pressure. This causes an increase in the contact area and the adhesion between the textile and blood. Blood penetrates the fibers and presents a Wenzel state on the hydrophilic polyester textile, causing the highest adhesion force of the textile on dry blood (Figure 3g).

### Low Adhesion of Bio-Fluids on the Textile

**Figure 4:**
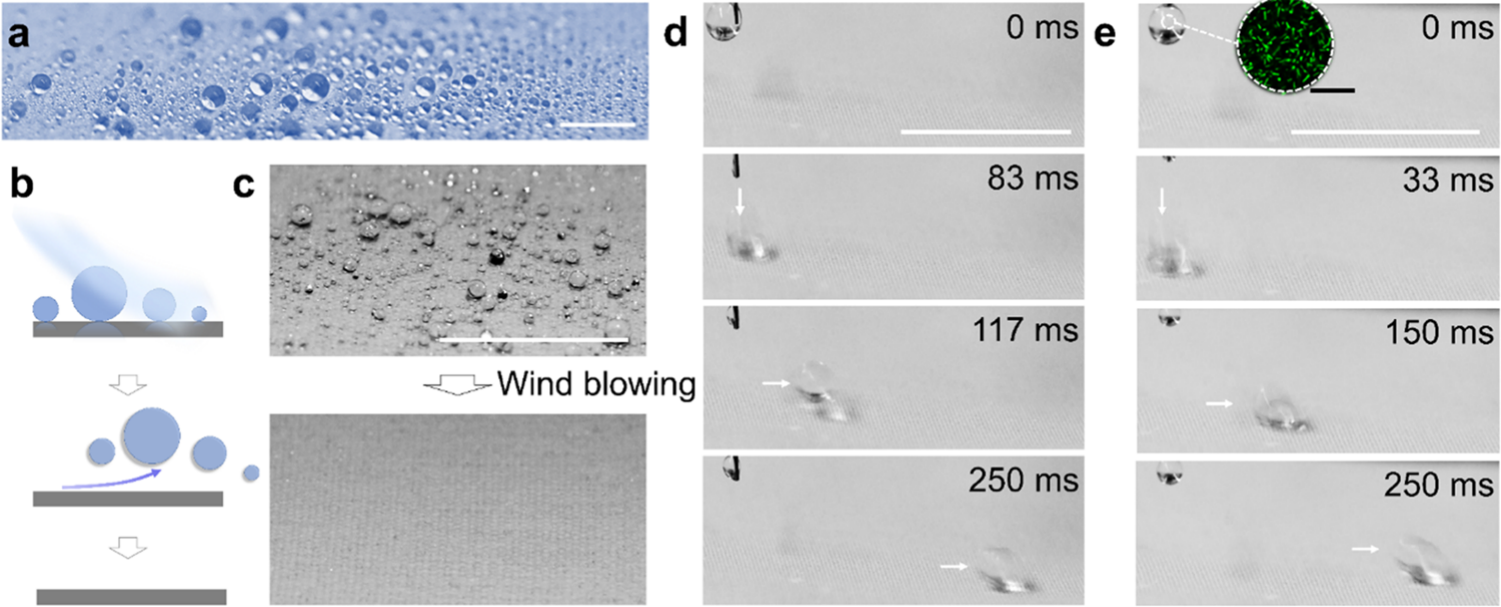
Repellency of the TiO_2_/PDMS-coated textile to bio-fluids. (a) Digital image shows spherical shapes of small droplets of water on the TiO_2_/PDMS-coated textile. Scale bar: 2 mm. (b) Schemes illustrate the detachment of droplets on the superhydrophobic surface by wind. (c) Detachment of small droplets of water under breeze (5 m/s). Scale bar: 1 cm. (d) Image series show sliding of a saliva droplet on the tilt TiO_2_/PDMS-coated textile. Tilt angle: α = 3°. Scale bar: 1 cm. (e) Image series show sliding of an *E. coli* bacterial solution droplet on the tilted TiO_2_/PDMS-coated textile. Tilt angle: α = 3°. Scale bar: 1 mm. The inset shows the image of *E. coli* bacteria dispersed in phosphate buffer saline, which was taken with a laser scanning confocal microscope. The density of the bacteria is: OD_600_ = 0.1. Scale bar: 20 μm.

As a further test of the low adhesion with respect to water, we deposited small droplets (*D* < 1 mm and V < 0.5 μL, *D* and *V* are droplet diameter and volume) on a horizontally oriented TiO_2_/PDMS-coated textile (Figure 4a). The small droplets were sprayed on the surface by an ultrasonic humidifier. These small, spherical droplets could be rapidly removed by an air breeze with a velocity of 5 m/s (Figure 4b,c). It implies that the small droplets can also easily detach from the TiO_2_/PDMS-coated textile surface, e.g., by shaking or vibrations.

Several bio-fluids were taken as examples to investigate the low adhesion effect. A saliva droplet (5 μL, 50 wt %) was placed and pressed on the TiO_2_/PDMS-coated textile with a syringe needle (Figure S3a). After holding for 3 s, the saliva droplet was completely detached without stains. In addition, the saliva droplet (7.3 μL) rolled off the coated textile easily at a low tilt angle (α = 3°, Figure 4d). The textile was further investigated for its repellency to bacterial solution. Here, we use *E. coli* as an example (Figure 4e). At a low tilt angle of α = 3°, a drop (7.3 μL) of bacterial solution (OD_600_ = 0.1) rolls off the surface without attachment. Various fluids such as vinegar, wine, milk, and coffee all present spherical shapes on the coated textile (Figure S3b). Not only that but also the coated textile effectively prevents sauces such as tomato sauce from sticking (Figure S3c). The low adhesion of kitchen fluids or sauces can effectively reduce the risk of surface contamination.

### Mechanical and Chemical Robustness

**Figure 5:**
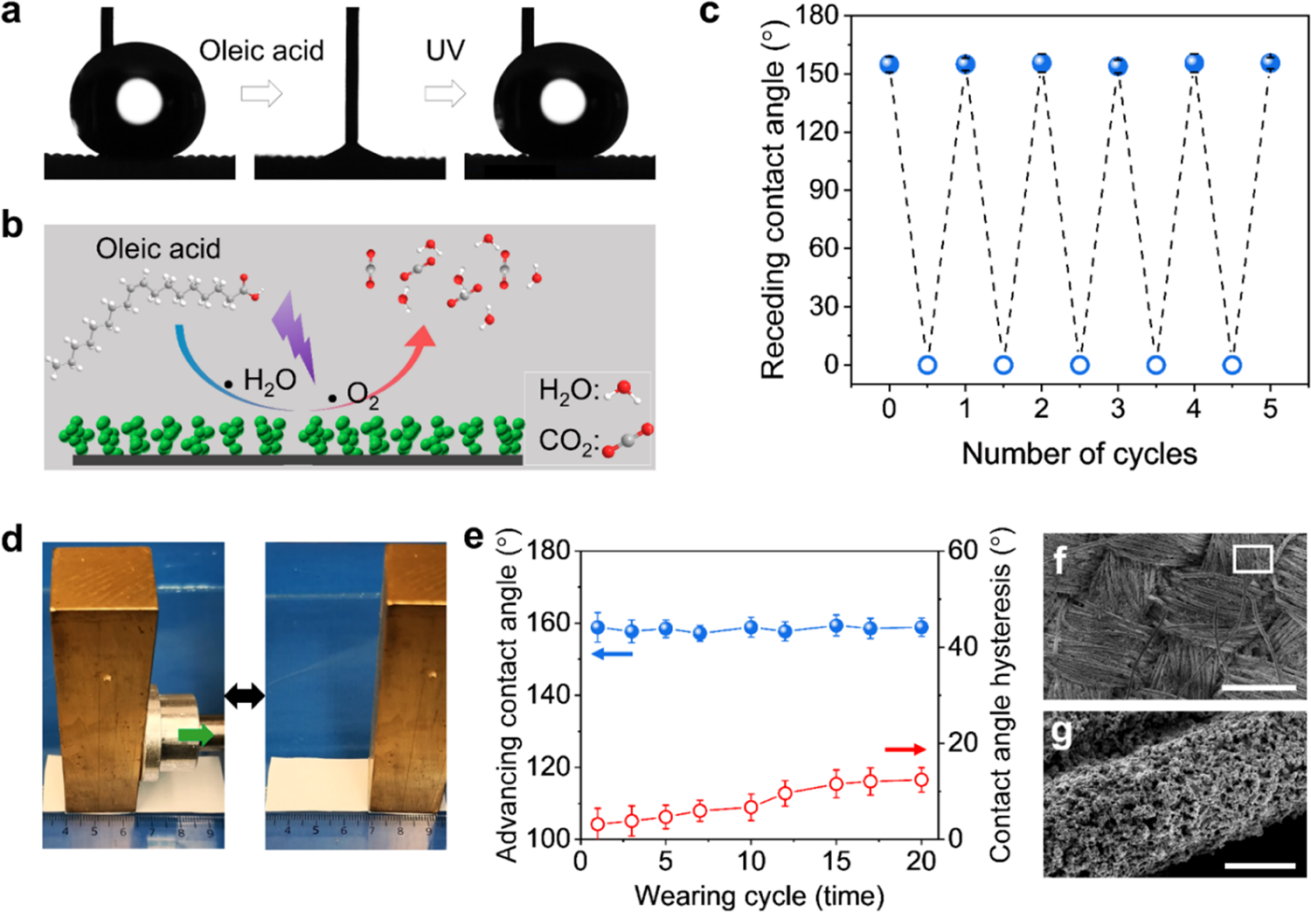
Chemical and mechanical robustness of the TiO_2_/PDMS-coated textile. (a) Images of the receding contact angle of water on the TiO_2_/PDMS-coated textile before (left) and after oleic acid contamination (middle), as well as after UV-A illumination (10 mW/cm^2^) for 30 min (right). (b) Scheme illustrates the degradation of oleic acid on the TiO_2_/PDMS film under UV illumination. The chemical is degraded to water and carbon dioxide at last. (c) Diagram shows the variation of the receding contact angle of water drops on the TiO_2_/PDMS film via oleic acid contamination and UV-A illumination for 5 cycles. (d) Wearing test of the TiO_2_/PDMS-coated textile with a sandpaper. The wearing process was always along the direction as shown. The mesh of the sandpaper is 400, and the mean structure size is around 25 μm. (e) Advancing contact angle and contact angle hysteresis of water on the TiO_2_/PDMS-coated textile wear with 400 mesh sandpaper under a pressure of 8 kPa. (f, g) SEM images show the surface morphology of the TiO_2_/PDMS-coated textile after wearing for 10 cycles with different magnifications. Scale bar: 200 μm (f), 5 μm (g).

Chemical and mechanical robustness of the coatings determine their long-term performance.^28,29^ Due to the chemical stability of PDMS, the TiO_2_/PDMS coatings are not degraded by UV-A light illumination (Figure S4). The TiO_2_ nanoparticles endow the surface with photocatalytic activity.^30^ When the coated textile is contaminated by surfactants such as oleic acid, the liquid repellent performance is lost. Generating radicals on the surface illuminated by UV light^31−33^ leads to degradation of chemicals on the surface (Figure 5a,b). Using the photocatalytic activity of the PDMS-coated TiO_2_ nanoparticles, superhydrophobicity was recovered after UV-A illumination (10 mW/cm^2^). According to our previous study, the photocatalytic activity also promotes the killing of attached bacteria.^23^ After five cycles of contamination using oleic acid and successive photo-degradation, the PDMS/TiO_2_ textile remained superhydrophobicity with a receding contact angle larger than 150° (Figure 5c).

Our TiO_2_/PDMS-covered textiles resist even strong treatment with sandpaper (mesh: 400, mean structure size is around 25 μm) loaded with a pressure of 8 kPa (Figure 5d). The fibers of the textile can deliver resistance to abrasion; the intermittent nanostructures in the pores between the microfibers ensure a stable superhydrophobicity.^34,35^ The advancing contact angle of the textile remains to be 157° ± 5° independent of wearing cycles (Figure 5e). As shown in Figure 5f, the fibers were broken during the wearing process. Nevertheless, the TiO_2_/PDMS nanostructures can firmly adhere to the fibers even though they are broken (Figure 5g). With more wearing cycles, the contact angle hysteresis of water on the TiO_2_/PDMS-coated surface increased from 3° to 10° after 15 wearing cycles. The increase in the contact angle hysteresis is caused by the increased number of flexible broken fibers, the section of which has no nanostructures covered and is hydrophilic.

### Flame-Retardant Performance

**Figure 6:**
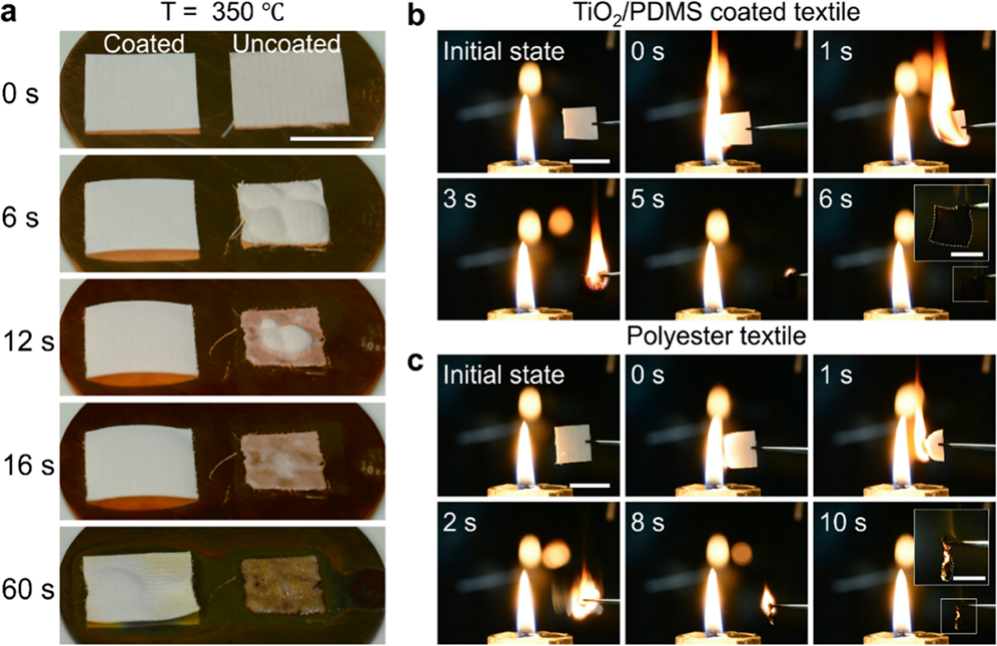
High-temperature resistance and flame-retardant effect. (a) Image series shows the change of the textile placed on a 350 °C hot copper block. Left sample: TiO_2_/PDMS-coated polyester textile. Right sample: bare polyester textile. Scale bar: 1 cm. (b) Images show the burning of the TiO_2_/PDMS-coated textile (1 × 1 cm^2^) lit by a candle flame. Scale bar: 1 cm. (c) Images show the burning of the polyester textile (1 × 1 cm^2^) lit by a candle flame. Scale bar: 1 mm. The insets in (b, c) show the final morphology of the textile after burning. Scale bar: 0.5 cm.

Two pieces of textiles, one coated and another uncoated with the TiO_2_/PDMS nanostructure, were placed on the same copper plate at a temperature of 350 °C, which is higher than the melting temperature (*T*_m_: 220–225 °C) of polyester (Figure 6a). The coated textile maintained a square shape with a slight change from the original shape even after 60 s. In contrast, when the uncoated textile touched the copper plate, it started to shrink rapidly at *t* = 6 s. It is the dense nanostructure coated on the fibers that blocks the direct contact between the textile and the substrate, which inhibits heat transfer from the heat plate to the textile and reduces shrinkage of the textile after melting. After that, the textile melted and attached to the plate (*t* = 16 s). It became scorched with brown color and boiled at *t* = 60 s. When the textiles were ignited with a candle flame, the fire on the coated textile spread from one side to another at a smooth pace (Figure 6b). For a 1 × 1 cm^2^ textile, the burning time was 6 s, after which the residue kept a square shape. When the uncoated textile was ignited, it shrank rapidly (*t* = 1 s) and then burned violently (*t* = 2 s) (Figure 6c). As shown in the image taken at *t* = 8 s, the burning polymer tends to drop down, which is one of the main reasons for the spread of flames in real life. The burning of the uncoated textile with a size of 1 × 1 cm^2^ resisted 10 s. From this, it is evident that the TiO_2_/PDMS-coated textile can efficiently stop flames from burning and spreading.

## Conclusions

In summary, a robust superhydrophobic textile coated with the TiO_2_/PDMS nanostructure was prepared. On such textiles, dried blood droplets can be removed from the surface by gravity alone. By comparing the peeling forces of the dried blood on textiles with different wetting properties, we found that the Cassie state of the dry blood is critical for reducing its adhesion to the surface. The stable superhydrophobicity decreases the adhesion of bio-fluids such as salvia and concentrated bacteria solutions to the surface, thereby reducing the risk of infections. Combined with the outstanding durability to keep its superhydrophobicity under wear and flame-retardant property, the TiO_2_/PDMS-coated textile has significant potential to be used as wound dressing, protective clothing, masks, firefighting clothes, etc.

## Experimental Section

### Modification of Titanium Dioxide Nanoparticles

Titanium dioxide nanoparticles (TiO_2_, 0.5 g, diameter: 21 ± 5 nm, P25, Sigma) were dispersed in tetrahydrofuran (THF, 10 mL) by sonication and mechanical stirring. Polymethylhydrosiloxane (PMHS, 30 mL, *M*_w_: 1.5–3.2 kDa, Gelest Inc.) was added and mixed uniformly with TiO_2_ nanoparticles. After the THF was evaporated for 24 h, the particle dispersion was illuminated with UV-A light (intensity: 10 mW cm^–2^) under stirring for 10 h to covalently link the PMHS to the TiO_2_.^31^ Afterward, the modified TiO_2_ nanoparticles were purified by centrifugation at 10,000 rpm for 10 min and redispersed in toluene. This process was repeated three times. The modified nanoparticles easily disperse in organic solvents such as toluene, THF, and n-hexane. Finally, we prepared dispersions with a 7 wt % concentration. The concentration of modified TiO_2_ nanoparticles dispersed in toluene was measured by weighing the deposition of 10 μL of dispersion with a balance (Sartorius Genius ME, Mettler Toledo) after evaporation of the solvent. 1*H*,1*H*,2*H*,2*H*-Perfluorooctyltrimethoxysilane (Sigma) was used to prepare the fluorinated textile. After treatment with oxygen plasma, the polyester textile was deposited with a layer of fluorosilane through chemical vapor deposition (CVD), and then the textile was heated at 120 °C for 2 h.

### Preparation of the TiO_2_/PDMS-Coated Textile

The modified TiO_2_ nanoparticles were mixed with the vinyl-terminated PDMS (vinyl-PDMS, *M*_w_: 62.0 kDa, Gelest) (*m*_PMHS-TiO2_/*m*_V-PDMS_ = 0.1) as well as a Pt-catalyst (0.005 wt % relative to vinyl-PDMS, platinum(0)-1,3-divinyl-1,1,3,3-tetramethyldisiloxane complex solution in xylene, Gelest) at a certain ratio in silicone oil (SO, viscosity: 10 cSt; *V*_toluene_/*V*_V-PDMS_ = 3). We modified the polyester fabrics to be superhydrophilic with oxygen plasma (5 min, power: 100%). Then we immersed the substrates in the mixture and allowed it to react for 6 h at 60 °C. The cross-linking reaction between PDMS molecules occurred both in bulk and on the substrates’ surfaces. After washing the samples with toluene to remove PDMS residues, superhydrophobic nanostructures formed on the surface. The surface morphology was characterized with a scanning electron microscope (SEM, 1530 Gemini LEO, Zeiss).

### Human Blood

Human blood was obtained from the Department of Transfusion Medicine Mainz from 10 healthy donors after a physical examination and after obtaining their informed consent in accordance with the Declaration of Helsinki. The use of human blood was approved by the local ethics committee “Landesärztekammer Rheinland-Pfalz” (837.439.12 (8540-F)). The blood contains heparin to prevent clotting.

### Peeling Force Measurement

100 microlitres of human blood was dropped on glass surfaces and pretreated with oxygen plasma. Then the polyester textile, fluorinated textile, and TiO_2_/PDMS-coated textile were covered on top under a load of 7.2 kPa. The water part of the blood completely evaporated after placement for 12 h at a room temperature of 25 °C and a relative humidity of 30%. The peeling forces between textiles and dry blood were measured via a Universal Testing Machine (Zwick/Roell Z005).

### Bio-Fluids Repellency Test

Water microdroplets were produced via a humidifier. After spraying the artificial fog on the TiO_2_/PDMS-coated textile surface, microdroplets with a broad size distribution formed on the surface. Saliva was collected from two healthy donors. *E. coli* K12 MG1655 was transformed with a fluorescent protein expression plasmid (GFP-pTrc99A, ampicillin-resistant, isopropyl β-d-thiogalactoside (IPTG) inducible). 10 mL of Lysogeny broth (LB) medium containing 50 μg/mL ampicillin and 0.5 mM IPTG was inoculated with a single colony and incubated overnight at 37 °C at 250 rpm. The bacteria were diluted with phosphate buffer saline (PBS) or LB medium, to the desired density (OD_600_ = 0.1). Bacteria were observed by using a Leica SP8 laser scanning confocal microscope with an excitation wavelength of 488 nm. All bacteria displayed green fluorescence due to the expression of the GFP protein. All of the photos or videos of bio-fluid motion on the TiO_2_/PDMS-coated textile were taken by a digital camera (Sony FE 90 mm f/2.8 Macro G OSS Lens).

### Wear Test

A sandpaper (400 mesh, 2.6 × 2.6 cm^2^) was fixed on a 540 g copper block with a double-sided tape. The pressure applied to this area was 8 kPa. The sandpaper was placed face-down onto the TiO_2_/PDMS-coated textile. The wearing test was then carried out by horizontally moving the copper block. After a given time of abrasion for a length of 3 cm, the advancing and receding contact angles were measured to characterize the stable superhydrophobicity of the textile.

### Heat Treatment and Burning Test

The textiles were cut into pieces with a size of 1 × 1 cm^2^. A copper plate (thickness: 0.5 mm) was preheated to 350 °C. The coated and uncoated textiles were placed on the plate at the same time. For the burning test, textiles were ignited with a candle flame. The processes were recorded with a digital camera (Nikon D7100).

## Supporting Information Available

Shape evolution of a blood drop with time on the polyester textile (Figure S1); the shape of the dried blood drop on surfaces (Figure S2); anti-adhesion property of the TiO2/PDMS-coated textile (Figure S3), and stable superhydrophobicity of the TiO_2_/PDMS-coated textile under UV illumination (Figure S4) (PDF)
