# Supplementary material for: Mechanically Robust and Flame-Retardant Superhydrophobic Textiles with Anti-Biofouling Performance
Source: Langmuir. 2022 Oct 14;38(42):12961–7. doi: 10.1021/acs.langmuir.2c02248 (PMC9609305; doi:10.1021/acs.langmuir.2c02248)
Supplement: Supplementary file 1 — la2c02248_si_001.pdf [file la2c02248_si_001.pdf]

Supporting Information

Mechanically Robust and Flame-Retardant Superhydrophobic Textiles with Anti-Biofouling Performance

Jie Liu,[*,†] *Yuling Sun,*[*,†] *Rui Ma,*[‡] *Xiaoteng Zhou,*[†] *Lijun Ye,*[†] *Volker Mailänder,*[†,§] *Werner Steffen,*[†] *Michael Kappl,*[†] *Hans-Jürgen Butt*[*,†]

[†]Max Planck Institute for Polymer Research, Ackermannweg 10, D-55128, Mainz, Germany.

[‡]The Second Clinical Division of Peking University School and Hospital of Stomatology, Anlilu 66, 100101, Beijing, China.

[§]Department of Dermatology, University Medical Center of the Johannes Gutenberg-University Mainz, Langenbeckstr. 1, 55131 Mainz, Germany

KEYWORDS: superhydrophobic, liquid adhesion, textile, anti-fouling, flame retardant

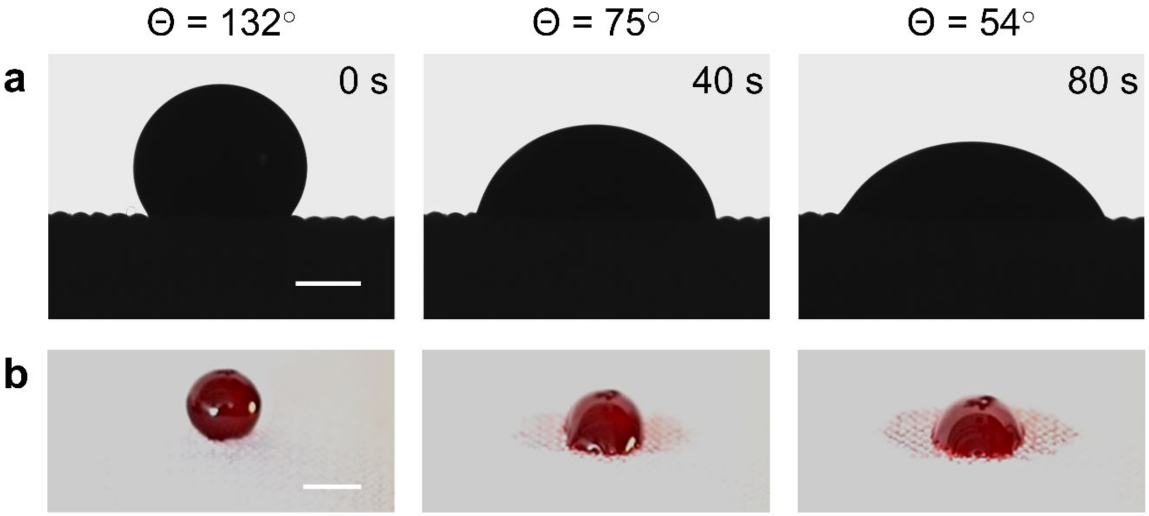

Figure S1. Shape evolution of a blood drop with time on the polyester textile. (a) The blood droplet (5 μL) on the uncoated polyester textile surface penetrates easily and rapidly into the pores between the fibers, indicated by the decreasing contact angle with time. Scale bar: 1 mm. (b) Digital images visually present the process in (a). The penetration and spreading of blood on the textiles will increase the blood adhesion strength and contamination area. Scale bar: 2 mm.

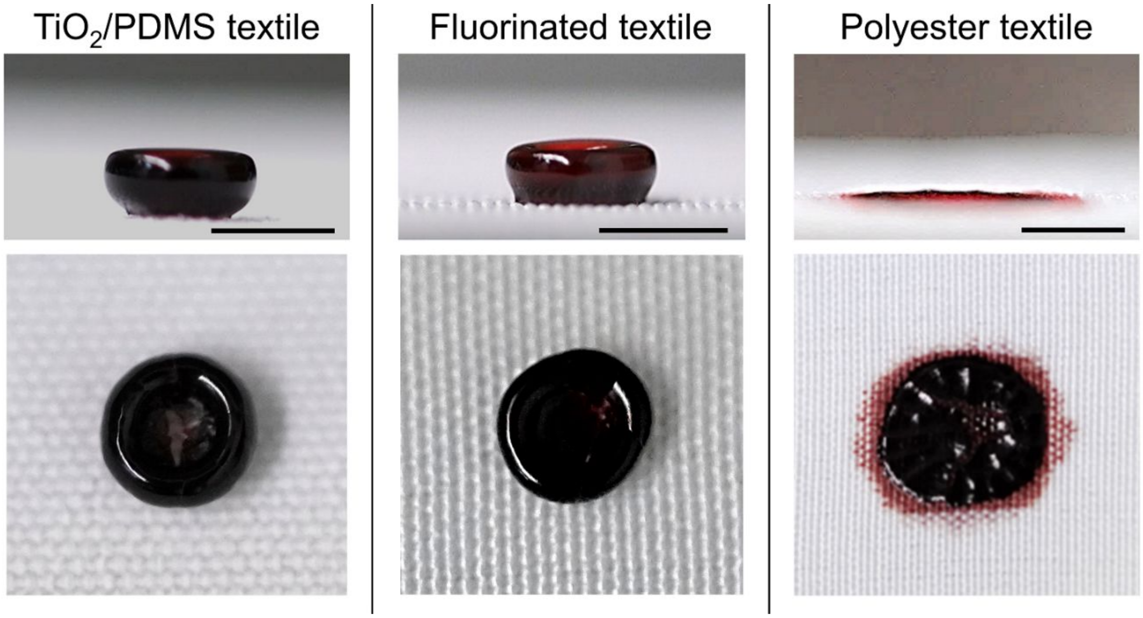

Figure S2. The shape of the dried blood drop on surfaces. On the TiO_2/PDMS coated textile (left), the shape of the dried blood drop was a bowl-like structure with two high sides and a low center. The contact angle of the dry blood is around 152°. On the fluorinated textile (middle), the similar bowl-like shape of the fried blood drop was formed. However, the contact angle of the dried blood is around 123°. On the polyester textile (right), blood penetrated the pores between fibers and the dried blood fully spread on the surface. The final contact angle is 0°. The dried bloods were from evaporation of 20 μL blood drops. Scale bar (from left to right): 2 mm, 2 mm, and 4 mm.

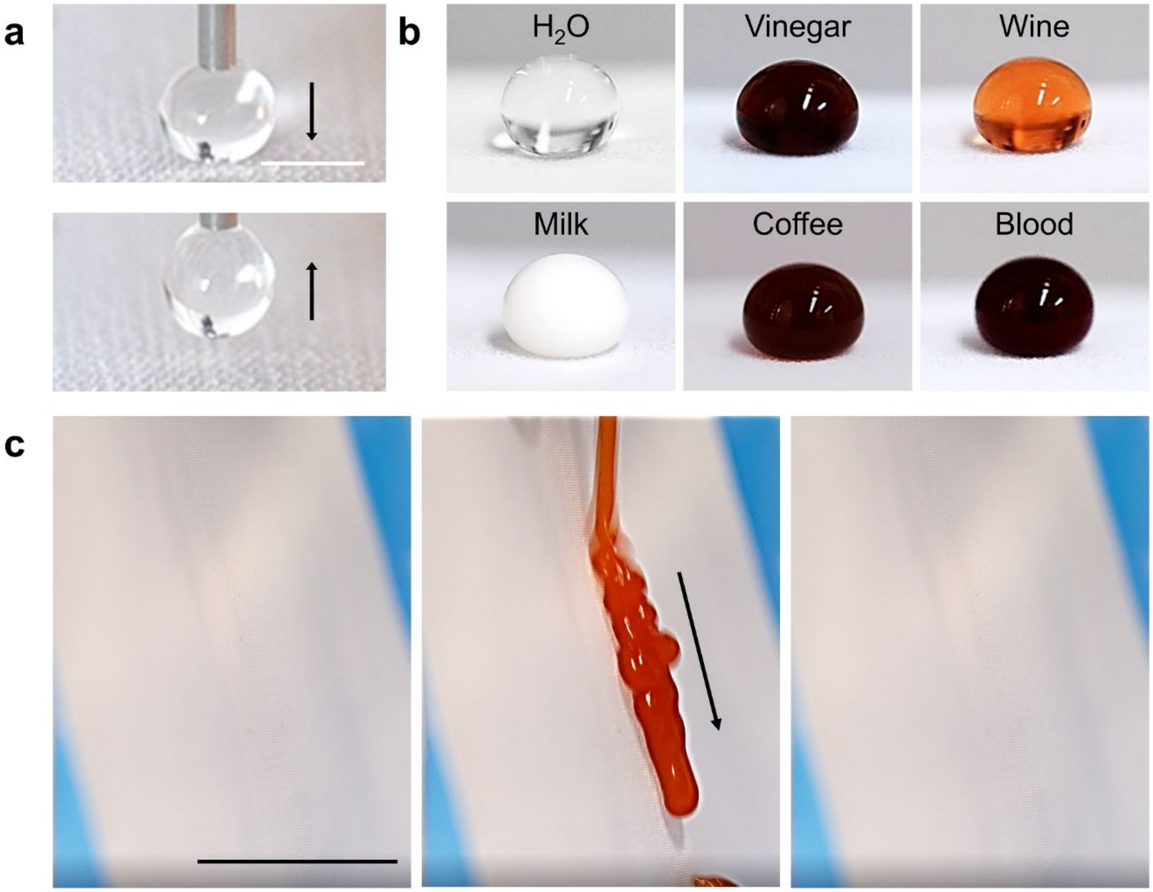

Figure S3. Anti-adhesion property of the TiO_2/PDMS coated textile. (a) A saliva droplet (5 μL, 50 wt%) was placed and pressed on the TiO_2/PDMS coated textile with a syringe needle. No stains were left on the textile. Scale bar: 2 mm. (b) Digital images show the drops of various fluids on the coated textile. All drops present spherical shapes on the surface. (c) Images show self-detachment of tomato sauce under gravity on the coated textile. No sauce stains were left on the surface. Scale bar: 1 mm.

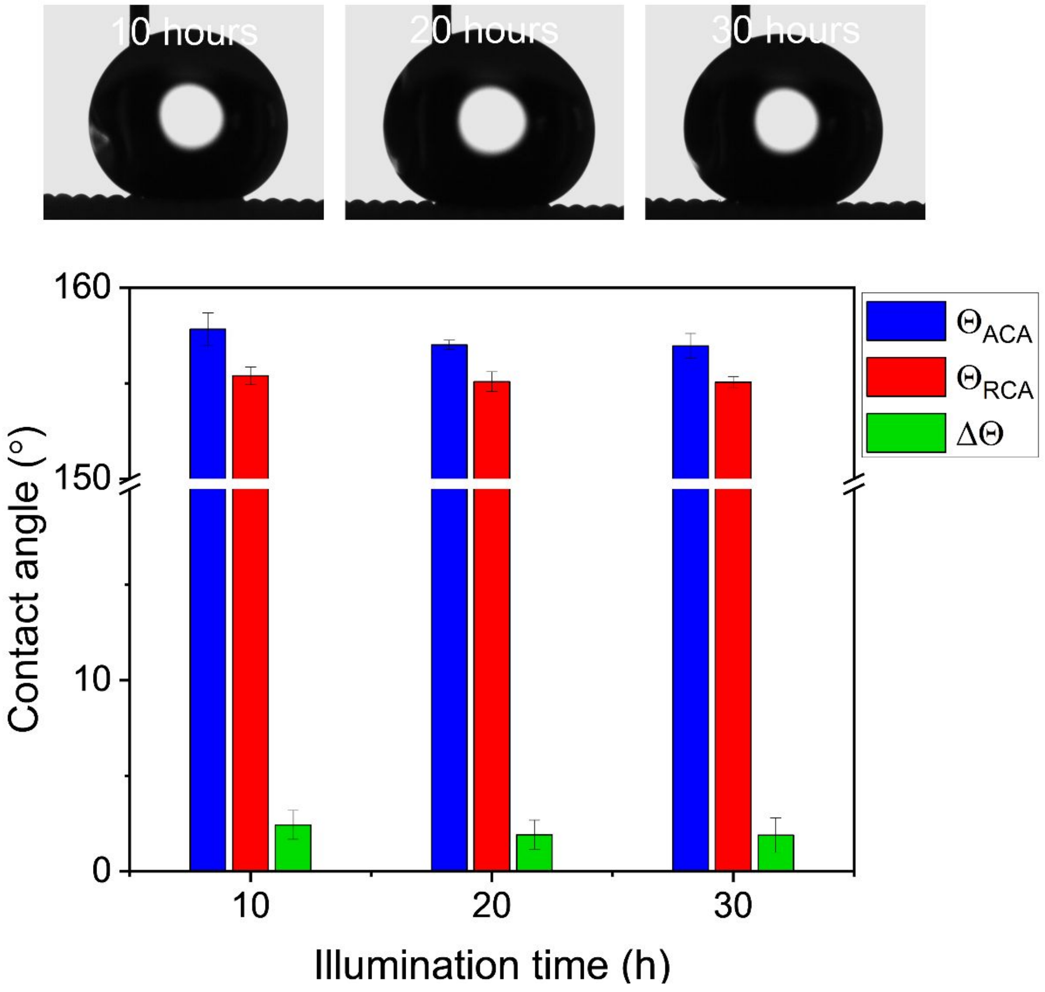

Figure S4. Stable superhydrophobicity of the TiO_2/PDMS coated textile under UV illumination. Images show the shapes of water droplets (5 μL) on the coated textiles illuminated with UV-A light (10 mW/cm^2) for different time: 10 h, 20 h, 30 h. The PDMS/TiO_2 textile remained superhydrophobicity with both the advancing (Θ_{ACA}) and receding (Θ_{RCA}) contact angles of water larger than 150° independent of illumination time. The contact angle hysteresis of water keeps constant with $\Delta\Theta = 2° \pm 1°$.
